# Essential oil yield and compositions of *Dracocephalum moldavica* L. in intercropping with fenugreek, inoculation with mycorrhizal fungi and bacteria

**DOI:** 10.1038/s41598-023-35156-x

**Published:** 2023-05-17

**Authors:** Zahra Amiriyan Chelan, Rouhollah Amini, Adel Dabbagh Mohammadi Nasab

**Affiliations:** Department of Plant Ecophysiology, Faculty of Agriculture, University of Tabriz, Tabriz, Iran

**Keywords:** Ecology, Plant sciences, Environmental sciences

## Abstract

Intercropping is one of the most important components of sustainable agriculture. The effects of chemical fertilizer (CF), arbuscular mycorrhizal fungi (AMF) (*Glomus* sp.) and AMF + nitrogen-fixing bacteria (NFB) including *Azospirillum* and *Azotobacter* (AMF + NFB) was studied on essential oil yield and compositions of Moldavian balm (Mb) (*Dracocephalum moldavica* L.) in sole cropping and intercropping with fenugreek (F) (*Trigonella foenum-graecum* L.). The experiment was conducted during 2020 and 2021 growing seasons in East Azarbayhan, Iran. The highest dry herbage yield (6132 kg ha^−1^) was obtained in Mb:F(4:2) and CF treatment. After sole Moldavian balm, the highest essential oil yield (15.28 kg ha^−1^) was obtained in Mb:F (4:2) and AMF + NFB treatment. Geranial, geranyl acetate, geraniol, neral, and nerol were the main chemical constituents of essential oil. In AMF + NFB treatments the geranial contents in intercropping patterns of Mb:F (1:1), (2:2) and (100:50), increased by 25.1, 15.5 and 34.6% compared with sole Moldavian balm. The highest LER_T_ values were observed in Mb:F (100:50) cropping pattern in 2021 (1.70 and 1.63 for CF and AMF + NFB treatments). Generally, it can be concluded that Mb:F (100:50) intercropping and use of AMF + NFB bio-fertilizer could be recommended to medicinal plant growers in sustainable production systems.

## Introduction

Conventional agricultural systems are designed in such a way which have reduced the existing plant diversity to a minimum, increased damage of pests and diseases, reduced quality of crops, severe soil erosion and loss of natural resources^1^. Intercropping of different species, cultivars or different isolates is one of the main components of sustainable agriculture^2^. In this cropping system, the goal is to increase the yield in terms of time and place and the plants use environmental resources with the highest efficiency^3,4^. Economic and environmental problems caused by the wasted of nitrogen fertilizers as a result of processes such as denitrification and nitrate leaching have led to the replacement of chemical fertilizers with organic and bio-fertilizers^5^. In sustainable production systems, bio-fertilizers could be used to reduce application of chemical fertilizers, support soil microbial activities and health and affect crop quality and yield^2,5^. It seems that the combined using of bio-fertilizers and intercropping could improve the production stability in arable lands^5,6^.

Moldavian balm (*Dracocephalum moldavica* L.) a medicinal plant in Lamiaceae family is domesticated in Central and Eastern Europe and native to Central Asia^2,7^. According to Amini et al.^8,9^ in Moldavian balm the geranial, geraniol, neral, and geranyl acetate are the main components of essential oil. Karimzadeh Asl et al.^10^ reported that use of bio-fertilizer in Moldavian balm increased the essential oil yield, geraniol and geranyl acetate contents. Fenugreek (*Trigonella foenum-graecum* L.) is one of the oldest known medicinal plants in the world, in Fabaceae family^11^. It is cultivated mostly in cold regions with moderate or low rainfall^12^. This medicinal plant has therapeutic properties and could be used as treatment of indigestion and diabetes, and as well as lowers blood pressure and cholesterol^13,14^. Medicinal plants are known as valuable resources for the production of medicinal products and providing health to communities^15^.

One of the most successful intercropping systems is the cultivation of legumes with non-legume crops, which in most cases causes the superiority of intercropping over sole crop^6^. In this cropping system, nitrogen stabilized by legumes is transferred to the accompanying crops and can contribute to yield stability in low-input agriculture^16^. Previous studies indicated that intercropping can improve the quantity and quality of medicinal plants^2,16^. Hence, the implementation of intercropping system of medicinal plants, one of its components is nitrogen fixative, can play a more effective role in using environmental resources and increase the productivity of cropping system^17^. Intercropping of Moldavian balm with faba bean (*Vicia faba* L.)^2^ and peppermint (*Mentha* × *piperita* L.) with soybean (*Glycine max* L.)^18^ improved the essential oil yield and affected its composition.

Arbuscular mycorrhizal fungi (AMF) are obligate biotrophs, forming symbiotic associations with the majority of vascular plants, including most crops^19^. The fungal mycelia colonize and interconnect roots of different crops, forming common mycorrhizal networks that bound to the outcomes of plant facilitative or competitive interactions and allow direct and efficient pathways of resource transfers between intercrops^20^. In a legume-based intercropping system, AMF are essentially needed to alleviate the nutrient shortages and support the extra P need for N fixation^21^. Also in intercropping systems, AMF have major roles such as mediation of plant interspecific transfer of C, N, P, and water resources and facilitative interactions^6^. Ingraffia et al.^22^ showed that AM fungi accounted for 20% of increases in the N fixed by faba bean (*Vicia faba*) and also in the N transferred to the intercropped wheat (*Triticum turgidum*). The nitrogen-fixing bacteria (NFB) Azospirillum and *Azotobacter* improve the nitrogen availability through converting the nitrogen into ammonia which is essential for crop growth^23^. Płaza et al.^24^ reported that the highest N and protein contents of spring barley (*Hordeum vulgare* L.) were observed in application of nitrogen-fixing bacteria (*Azotobacter chroococcum*) and intercropping with red clover (*Trifolium pratense* L.).

In sustainable production systems, application of bio-fertilizers in combination with intercropping reduce the chemical fertilizer application and minimize harmful impacts on agro-ecosystems. In previous researches the effects of intercropping and inoculation with AMF or NFB have been evaluated^6,24^ but there is a few studies about the co-inoculation of AMF and NFB in intercropping patterns. Intercropping with legume crop and combined inoculation with AMF and NFB could improve the essential oil yield and compositions of medicinal plants and affect the productivity of cropping system. So the aim of this study was investigating the effects of inoculation with AMF and NFB on essential oil yield and compositions of Moldavian balm at different intercropping patterns with fenugreek.

## Results

### Analysis of variance

The results of combined analysis of variance (ANOVA) (Table 1) indicated that the effects of year, cropping pattern and fertilizer treatment were significant (p≤ 0.01) on plant height, leaf area index, dry herbage yield, essential oil content and yield of Moldavian balm. The interaction effects of year × cropping pattern and year × fertilizer treatment on dry herbage yield (p≤ 0.05) and essential oil yield (p≤ 0.01) were significant. The interaction effect of year × cropping pattern was significant on plant height (p≤ 0.01). Also, plant height, leaf area index, dry herbage yield and essential oil yield of Moldavian balm were significantly affected by interaction effect of cropping pattern × fertilizer treatment (Table 1).

**Table 1:** The combined analysis of variance for effects of cropping pattern and fertilizer treatment on growth traits, essential oil content and yield of Moldavian balm.

Source of variation	df	Plant height	Leaf area index	Dry herbage yield	Essential oil content	Essential oil yield
Year (Y)	1	**	**	**	**	**
Block × Y	4	n.s	n.s	n.s	n.s	n.s
Cropping pattern (C)	4	**	**	**	**	**
Y × C	4	**	n.s	*	n.s	**
Fertilizer (F)	2	**	**	**	**	**
Y × F	2	n.s	n.s	*	n.s	**
C × F	8	*	*	**	n.s	*
Y × C × F	8	n.s	n.s	n.s	n.s	n.s
CV (%)	-	5.52	11.62	9.28	8.22	13.67

ns, * and **: non -significant and significant at P ≤ 0.05 and P ≤ 0.01, respectively.

### Plant height

**Figure 1:**
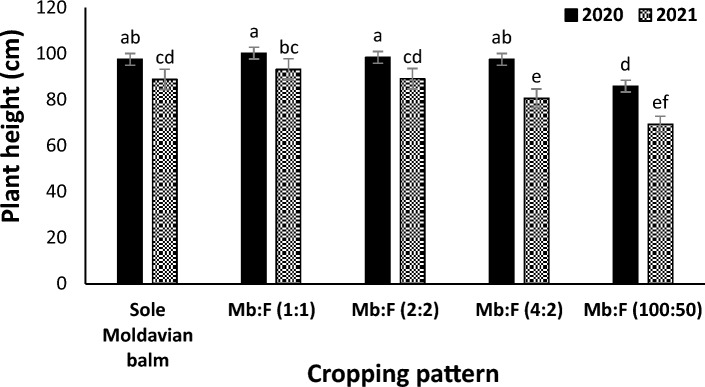
Plant height of Moldavian balm affected by interaction effect of year × cropping pattern. Different letters indicate significant differences at *p* ≤ 0.05.

**Figure 2:**
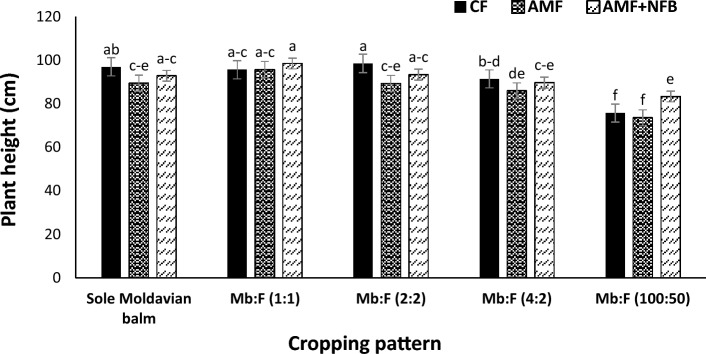
Plant height of Moldavian balm affected by interaction effect of cropping pattern × fertilizer treatment. Different letters indicate significant differences at *p* ≤ 0.05.

The mean comparison of interaction effect of year × cropping pattern (Fig. 1) showed that at all cropping patterns the plant heights in 2020 were greater than those in 2021 (13.5 and 28.2% reduction in 2020 and 2021, respectively, compared to sole Moldavian balm). In both years the plant height in Mb:F (100:50) cropping pattern decreased compared with those in sole Moldavian balm and other intercropping patterns (Fig. 1). The interaction of cropping pattern × fertilizer treatment indicated that in Mb:F(1:1), Mb:F(2:2) and Mb:F(4:2) cropping patterns and sole Moldavian balm the plant heights were not significantly different in CF and AMF + NFB treatments. In Mb:F(100:50) cropping pattern the plant height in AMF + NFB fertilizer treatment increased by 3.9 and 2.8% compared with that in CF and AMF treatment, respectively (Fig. 2).

### Leaf area index (LAI)

**Figure 3:**
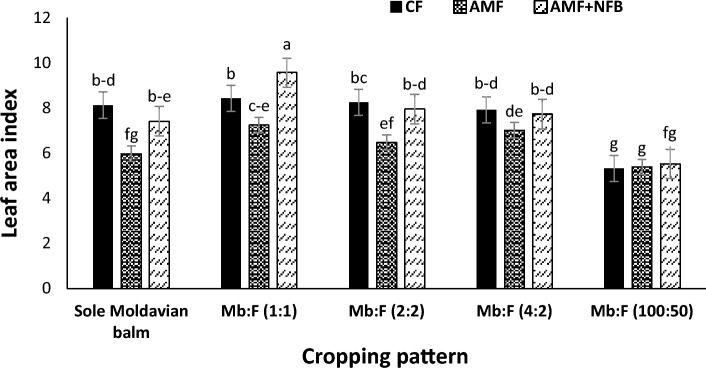
Leaf area index of Moldavian balm affected by interaction of cropping pattern × fertilizer treatment. Different letters indicate significant differences at *p* ≤ 0.05.

The mean comparison of LAI in two years showed that the LAI in 2020 (7.87) increased by 19.8% compared with 2021 (6.57) (Table 2). In intercropping pattern of Mb:F(1:1) the LAIs in AMF and AMF + NFB fertilizer treatments increased by 21.1 and 28.9%, respectively, compared with those in sole Moldavian balm and the highest LAI (9.56) was observed in AMF + NFB fertilizer treatment. In Mb:F(100:50) intercropping pattern, the lowest LAI was observed and the LAIs at all fertilizer treatments were not significantly different (Fig. 3).

**Table 2:** The effect of year, cropping pattern and fertilizer treatment on Moldavian balm height, leaf area index, dry herbage yield, essential oil content and yield. Mb: Moldavian balm, F: fenugreek. The cropping pattern including sole Moldavian balm (Mb), intercropping patterns of 1 row of Moldavian balm + 1 row of fenugreek (Mb:F (1:1)), 2 rows of Moldavian balm + 2 rows of fenugreek (Mb:F(2:2)), and 4 rows of Moldavian balm + 2 rows of fenugreek (Mb:F(4:2)) and additive intercropping of Moldavian balm + fenugreek Mb:F(100: 50). The fertilizer treatments were consisted of 100% chemical fertilizer (CF), application of arbuscular mycorrhizal fungi (AMF) and application of AMF + nitrogen-fixing bacteria (AMF + NFB). Means within each column with similar letter are not significantly different at *p* ≤ 0.05.

Effect	Levels	Plant height (cm)	Leaf area index	Dry herbage yield (kg ha^−1^)	Essential oil content (%)	Essential oil yield (kg ha^−1^)
Year	2020	95.88 ± 0.71 a	7.87 ± 0.125 a	5467 ± 68.75 a	0.261 ± 0.003 a	14.27 ± 0.248 a
	2021	84.17 ± 0.71 b	6.57 ± 0.125 b	4470 ± 68.75 b	0.226 ± 0.003 b	10.06 ± 0.248 b
Cropping pattern	Sole Moldavian balm	93.13 ± 1.17 b	7.17 ± 0.198 b	6819 ± 108.71 a	0.232 ± 0.005 cd	16.06 ± 0.392 a
	Mb:F (1:1)	96.66 ± 1.17 a	8.41 ± 0.198 a	3938 ± 108.71 d	0.252 ± 0.005 ab	10.11 ± 0.392 c
	Mb:F (2:2)	93.75 ± 1.17 ab	7.55 ± 0.198 b	3817 ± 108.71 e	0.261 ± 0.005 a	10.04 ± 0.392 c
	Mb:F (4:2)	89.06 ± 1.17 c	7.55 ± 0.198 b	5538 ± 108.71 b	0.245 ± 0.005 bc	13.72 ± 0.392 b
	Mb:F (100:50)	77.56 ± 1.17 d	5.41 ± 0.198 c	4744 ± 108.71 c	0.228 ± 0.005 d	10.89 ± 0.392 c
Fertilizer treatment	CF	91.67 ± 0.91 a	7.60 ± 0.153 a	5198 ± 84.21 b	0.237 ± 0.004 b	12.45 ± 0.304 b
	AMF	86.86 ± 0.91 b	6.42 ± 0.153 b	4343 ± 84.21 c	0.235 ± 0.004 b	10.13 ± 0.304 c
	AMF + NFB	91.56 ± 0.91 a	7.64 ± 0.153 a	5366 ± 84.21 a	0.259 ± 0.004 a	13.91 ± 0.304 a

### Dry herbage yield

**Figure 4:**
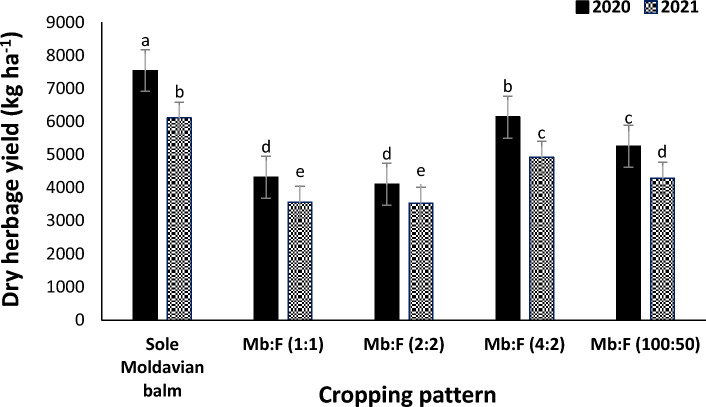
Dry herbage yield of Moldavian balm affected by interaction of year × cropping pattern. Different letters indicate significant differences at *p* ≤ 0.05.

**Figure 5:**
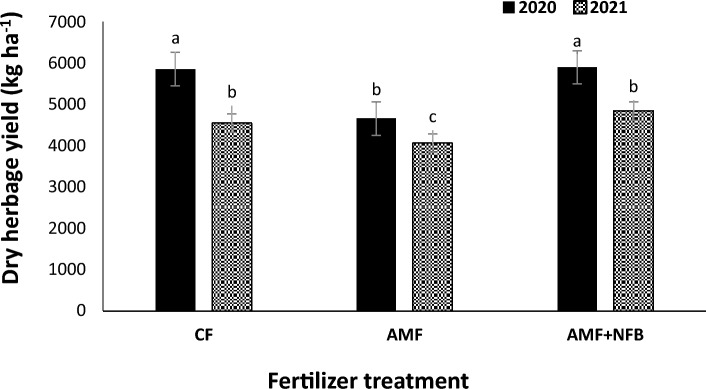
Dry herbage yield of Moldavian balm affected by interaction of year × fertilizer treatment. Different letters indicate significant differences at p ≤ 0.05.

**Figure 6:**
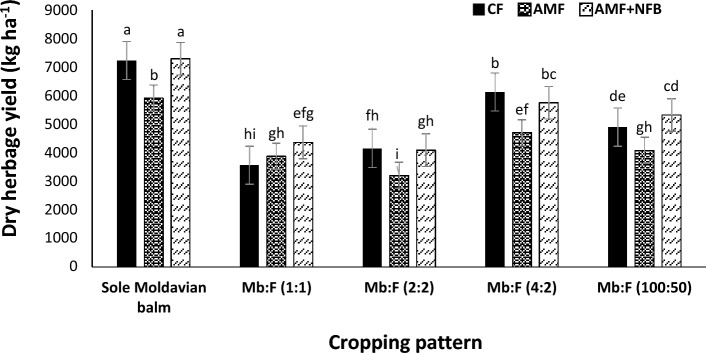
Dry herbage yield of Moldavian balm affected by interaction of cropping pattern × fertilizer treatment. Different letters indicate significant differences at *p* ≤ 0.05.

Moldavian balm dry herbage yield in 2020 (5467 kg ha^−1^) increased by 22.1% compared with than that in 2021 (4480 kg ha^−1^) (Table 2). The mean comparison for interaction effect of year × cropping pattern (Fig. 4) indicated that at all cropping patterns, the dry herbage yields in 2020 were greater than those in 2021. Among the intercropping patterns the highest dry herbage yield was obtained in Mb:F(4:2) cropping pattern. The results of interaction effect of year × fertilizer treatment (Fig. 5) indicated that dry herbage yield in AMF + NFB treatment was not significantly different with that in CF treatment. The mean comparison for interaction effect of cropping pattern × fertilizer treatment showed that at all intercropping patterns the dry herbage yield decreased compared with that in sole Moldavian balm (Fig. 6). The highest dry herbage yields were observed in Mb:F(4:2) intercropping pattern. In Mb:F(100:50) intercropping pattern the highest dry herbage yield (5322 kg ha^−1^) was observed in AMF + NFB treatment, while in Mb:F(4:2) intercropping pattern the highest dry herbage yield (6132 kg ha^−1^) was observed in CF treatment.

### Essential oil content (%)

The mean comparison indicated that the Moldavian balm essential oil content in 2020 (0.261%) increased by 15.5% compared with that in 2021 (0.226%) (Table 2). The highest essential oil content (0.261%) was obtained in Mb:F(2:2) intercropping pattern that was not significantly different with that in Mb:F(1:1). The lowest essential oil contents (0.228 and 0.232%) were obtained in Mb:F(100:50) and sole Moldavian balm, respectively. The essential oil content in AMF + NFB fertilizer treatment increased by 9.3 and 15.1% compared with those in CF and AMF fertilizer treatments, respectively (Table 2).

### Essential oil yield

**Figure 7:**
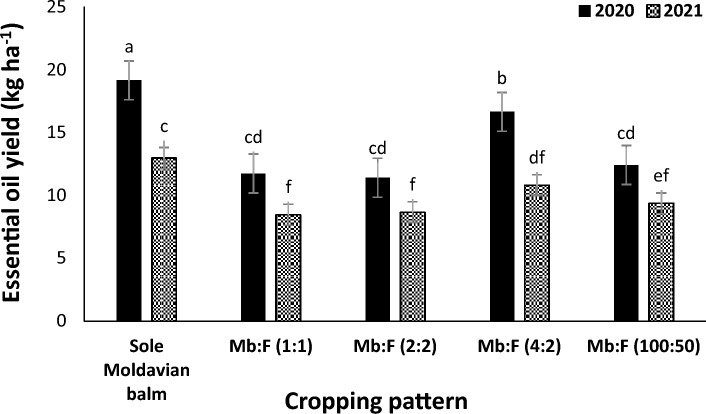
Essential oil yield of Moldavian balm affected by interaction of year × cropping pattern. Different letters indicate significant differences at p ≤ 0.05.

**Figure 8:**
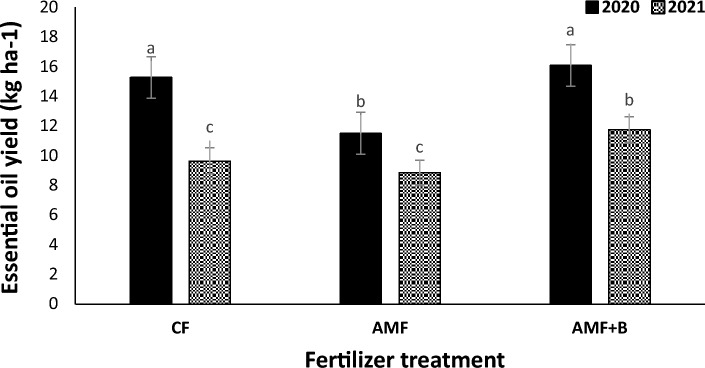
Essential oil yield of Moldavian balm affected by interaction of year × fertilizer treatment. Different letters indicate significant differences at p ≤ 0.05.

**Figure 9:**
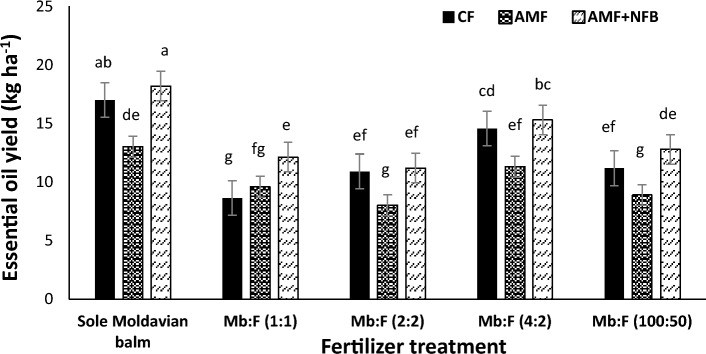
The interaction effect of cropping pattern × fertilizer treatment on essential oil yield of Moldavian balm. Different letters indicate significant differences at *p* ≤ 0.05.

The mean comparison (Fig. 7) indicated that at all cropping patterns the essential oil yield in 2020 were higher than those in 2021. The greatest essential oil yield was observed in Mb:F(4:2) intercropping pattern. The mean comparison of interaction effect of year × fertilizer treatment (Fig. 8) indicated that in 2020 the essential oil yield in AMF + NFB treatment was not significantly different with that in CF treatment and in 2021 the essential oil yield in AMF + NFB treatment increased by 21.9% compared with that in CF treatment. The mean comparison of interaction effect of cropping pattern × fertilizer treatment (Fig. 9) indicated that the essential oil yield decreased at all intercropping patterns compared with that in sole Moldavian balm. In Mb:F(2:2), Mb:F(4:2) and Mb:F(100:50) intercropping patterns the essential oil yield in AMF + NFB and CF treatment were not significantly different but in Mb:F(1:1) intercropping pattern, the essential oil yield in AMF + NFB treatment increased by 40.7% compared with that in CF treatment (Fig. 9).

### Essential oil composition (GC–MS)

Moldavian balm essential oil compositions was assessed through GC–MS analysis and it was found that the essential oil was consisted of 19 components, accounting for 98.3–99.2% of the total compounds (Table 3). Results of the GC–MS analysis indicated that the main essential oil constituents were geranial (6.3–61.4%), geranyl acetate (2.4–47.1%), geraniol (3.4–25.9%), neral (0.9–20.5%) and nerol (0.1–13.5%). The essential oil constituents affected differently by cropping pattern and fertilizer treatment. In sole Moldavian balm the highest geranial content (61.4%) was obtained in AMF bio-fertilizer treatment, increased by 46.9% compared with that in CF fertilizer (Table 3). In Mb:F(1:1) intercropping pattern the contents of geranial in AMF and AMF + NFB treatments increased by 9.0 and 12.8%, respectively, compared with CF fertilizer treatment, while in Mb:F(2:2) intercropping pattern the contents of geranial decreased in AMF and AMF + NFB treatments. In sole Moldavian balm the geraniol contents in AMF and AMF + NFB fertilizer treatments decreased by 79.8 and 42.6%, respectively, compared with that in CF fertilizer treatment (Table 3). In Mb:F(1:1) and (2:2) intercropping patterns the geraniol contents increased in AMF and AMF + NFB fertilizer treatments. The highest content of geranyl acetate (47.1%) was observed in Mb:F(2:2) intercropping pattern and AMF + NFB fertilizer treatment which increased by 276.8% compared with that in sole Moldavian balm. In sole Moldavian balm and Mb:F(4:2) intercropping pattern the contents of geranyl acetate in AMF and AMF + NFB fertilizer treatments increased compared with those in CF fertilizer treatments; while in intercropping patterns of Mb:F(1:1) and (2:2) the contents of geranyl acetate decreased in AMF and AMF + NFB fertilizer treatments. In all intercropping patterns and sole Moldavian balm the contents of neral in AMF and AMF + NFB fertilizer treatments improved in comparison with those in CF fertilizer treatments. The highest content of nerol (13.5%) was observed in sole Moldavian balm and CF fertilizer treatment. In sole Moldavian balm and intercropping patterns of Mb:F(2:2) and (4:2) the nerol contents in AMF and AMF + NFB fertilizer treatments decreased compared with those in CF fertilizer treatments (Table 3).

**Table 3:** Proportion of Moldavian balm essential oil constituents under different cropping patterns and fertilizer treatments (an average over the two years). The cropping pattern including sole Moldavian balm (Mb), intercropping patterns of 1 row of Moldavian balm + 1 row of fenugreek (Mb:F (1:1)), 2 rows of Moldavian balm + 2 rows of fenugreek (Mb:F(2:2)), and 4 rows of Moldavian balm + 2 rows of fenugreek (Mb:F(4:2)) and additive intercropping of Moldavian balm + fenugreek Mb:F(100: 50). The fertilizer treatments were consisted of 100% chemical fertilizer (CF), application of arbuscular mycorrhizal fungi (AMF) and application of AMF + nitrogen-fixing bacteria (AMF + NFB).

Compounds	RI^a^	Sole Moldavian balm (Mb)				Mb:F (1:1)				Mb:F (2:2)				Mb:F (4:2)				Mb:F (100:50)		
		CF	AMF	AMF + NFB		CF	AMF	AMF + NFB		CF	AMF	AMF + NFB		CF	AMF	AMF + NFB		CF	AMF	AMF + NFB
4-Hexen-1-ol,	879	–	–	–		–	–	–		–	–	–		–	–	–		0.5	0.5	–
1-Octen-3-ol	975	–	–	–		0.5	–	–		–	–	0.8		–	–	–		–	–	–
6-Methyl-5-hepten-2-one	983	–	–	–		1.7	–	–		0.4	1.2	1.2		1.1	–	–		–	0.7	–
Benzaldehyde	998	4.8	–	0.3		–	–	–		–	–	0.5		–	–	–		–	–	–
Linalool oxide	1087	–	–	–		0.7	–	–		–	–	1.2		–	–	–		–	–	–
Linalool	1104	–	–	–		1.5	–	–		0.9	0.1	2.9		0.4	1.5	–		–	1.1	0.3
**Nerol**	1235	**13.5**	**0.6**	**3.4**		**2.5**	**2.8**	**6.7**		**3.7**	**3.2**	**0.1**		**3.1**	**2.2**	**0.5**		**2.9**	**11.3**	**1.9**
**Neral**	1242	**0.9**	**8.9**	**6.4**		**2.2**	**4.6**	**3.3**		**3.2**	**16.2**	**4.4**		**8.1**	**20.5**	**18.1**		**6.0**	**14.5**	**17.7**
Linalyl formate	1258	5.2	–	5.4		–	0.7	5.9		–	5.8	–		–	–	–		–	–	–
**Geraniol**	1263	**25.8**	**5.2**	**14.8**		**8.1**	**26.2**	**25.9**		**13.5**	**21.4**	**14.2**		**20.5**	**9.2**	**25.8**		**21.7**	**25.8**	**3.4**
**Geranial**	1275	**41.8**	**61.4**	**43.1**		**47.8**	**52.1**	**53.9**		**51.7**	**26.3**	**50.2**		**37**	**43.1**	**6.3**		**46.8**	**15.8**	**58**
Geranyl formate	1305	–	12.2	11.5		0.1	–	–		–	–	0.4		20.9	–	–		10.4	–	7.1
Neryl acetate	1367	3.5	–	0.8		0.5	–	–		–	–	4.0		–	–	–		–	2.1	0.4
**Geranyl acetate**	1386	**3.1**	**10.2**	**12.5**		**31.2**	**12.3**	**2.4**		**25.3**	**23.5**	**17.4**		**7.7**	**21.8**	**47.1**		**9.1**	**26.3**	**8.8**
Farnesol	1722	–	–	0.2		0.5	-	1.1		–	0.2	0.2		–	–	0.8		1.0	–	0.1
Hexadecanoic acid (CAS)	1947	–	–	–		0.2	–	–		–	–	0.5		–	–	–		–	0.3	–
Di-(2-ethylhexyl)phthalate	2480	–	–	–		1.3	–	–		–	1	0.3		–	–	–		–	–	0.8
Total identified (%)		98.6	98.5	98.4		98.8	98.7	99.2		98.7	98.9	98.3		98.8	98.3	98.6		98.4	98.4	98.5

^a^RI: Retention Indices on the HP-5 MS column.

Significant values are in bold.

### LER

In both years the LER_T_ indices of all intercropping patterns were higher than 1.0 (Table 4). The highest LER_T_ values were obtained in Mb:F (100:50) intercropping patterns and CF fertilizer treatments (1.58 and 1.70 in 2020 and 2021, respectively ) and the lowest values in Mb:F(4:2) and AMF treatment (1.03 and 1.04 in 2020 and 2021, respectively) (Table 4). Among the replacement intercropping patterns the LER_T_ values in intercropping patterns of Mb:F(1:1) and (2:2) were higher than that of Mb:F(4:2). In Mb:F(1:1) and (2:2) intercropping patterns the highest LER_T_ values were observed in AMF + NFB fertilizer treatment and in Mb:F(100:50) intercropping pattern the highest LER_T_ values was observed in CF treatment. Comparison of LER_Mb_ and LER_F_ (partial LERs) in Mb:F(1:1) and (2:2) intercropping patterns showed that in most of the treatments the LER_F_ values were higher than LER_Mb_ values, which indicates that intercropping had more positive effect on fenugreek. In Mb:F(4:2) intercropping pattern the LER_Mb_ values were higher than those of LER_F_; while in Mb:F(100:50) intercropping pattern the LER_Mb_ values were lower than LER_F_ (Table 4).

**Table 4:** Land equivalent ratio (LER_T_) values at different intercropping patterns and fertilizer treatments in 2020 and 2021. Mb: Moldavian balm, F: fenugreek. LER_Mb_: LER of Moldavian balm, LER_F_: LER of fenugreek, LER_T_: total LER. CF, AMF and AMF + NFB are 100% chemical fertilizer, arbuscular mycorrhizal fungi and arbuscular mycorrhizal fungi + nitrogen-fixing bacteria, respectively.

Intercropping pattern	Fertilizer treatment		**2020**			**2021**	
		LER_Mb_	LER_F_	LER_T_	LER_Mb_	LER_F_	LER_T_
Mb:F (1:1)	CF	0.54	0.58	1.12	0.44	0.75	1.19
	AMF	0.65	0.58	1.23	0.67	0.58	1.24
	AMF + NFB	0.56	0.68	1.24	0.66	0.71	1.37
Mb:F (2:2)	CF	0.56	0.72	1.28	0.59	0.66	1.25
	AMF	0.51	0.65	1.16	0.58	0.55	1.13
	AMF + NFB	0.56	0.75	1.31	0.58	0.67	1.26
Mb:F (4:2)	CF	0.87	0.22	1.09	0.85	0.26	1.11
	AMF	0.82	0.21	1.03	0.78	0.26	1.04
	AMF + NFB	0.79	0.25	1.04	0.81	0.24	1.05
Mb:F (100:50)	CF	0.65	0.93	1.58	0.72	0.98	1.70
	AMF	0.74	0.75	1.49	0.63	0.69	1.32
	AMF + NFB	0.72	0.83	1.55	0.76	0.87	1.63

## Discussion

The Moldavian balm height in 2020 was higher than that in 2021, which can be attributed to more precipitation in 2020 than 2021 (Table 5). It was found that the Moldavian balm height increased in Mb:F(1:1) and (2:2) intercropping patterns with application of CF and AMF + NFB fertilizers (Fig. 2). According to Yang et al.^17^ in shade conditions caused by intercropping, increasing plant height is expected by reducing the ratio of red to far red light (FR/R) and reducing the amount of photosynthetic active radiation. Increasing the plant height in intercropping conditions can be attributed to competition for more light interception and lack of light penetration through the canopy and non-decomposition of auxin hormone in these conditions^25^. Also, Ebwongu et al.^26^ in corn (*Zea mays* L.)- potato (*Solanum tuberosum* L.) intercropping observed that the height of potato increased in intercropping compared to sole cropping. Also, increasing plant height in intercropping with legumes can be associated with high nitrogen availability^27^. The Moldavian balm height decreased in Mb:F(100:50) additive intercropping pattern compared to sole crop, that could be attributed to competition between crops for water, nutrients and biospace as a result of increasing crop density in unit area^27^. Agegnehu et al.^28^ also reported that the faba bean height reduced significantly in intercropping with barley (*Hordeum vulgare* L.) due to interspecific competition. In Mb:F(100:50) intercropping pattern the greatest plant height was observed in AMF + NFB treatment that may be explained by better absorption of phosphorus and nitrogen which ultimately has led to improve the growth traits such as height^29^.

**Table 5:** Monthly total precipitation and mean temperature in 2020 and 2021 growing seasons in the experimental area.

	Year	May	June	July	August	September
Total precipitation (mm)	2020	14.23	2.03	3.3	1.27	0
	2021	12.71	0	1.2	0	0
Mean temperature (°C)	2020	20.2	25.5	29	26	24.8
	2021	22.5	28.7	29.5	29.1	24.3

The highest LAI was observed in Mb:F(1:1) cropping pattern with application of AMF + NFB bio-fertilizer. The increase in Moldavian balm LAI in this treatment could be explained by improving the nutrients (N + P) availability through bio-fertilizer (AMF + NFB) treatment and biological nitrogen fixation by fenugreek. Nitrogen is one of the most important nutrients which affects crop growth, especially the development of leaf area by affecting the size and longevity of each leaf^30^. Our results are in consistent with Yavas and Unay^31^ on intercropping of corn and legumes. Reedy & Willey^32^ also observed that in intercropping of pearl millet (*Pennisetum glaucum* L.) and peanut (*Arachis hypogaea*) the millet LAI increased significantly compared to sole crop. They concluded that the most important factor of this superiority was the positive role of peanut in increasing the soil fertility. Decrease in LAI of Moldavian balm in Mb:F(100:50) intercropping compared with other cropping patterns could be attributed to interspecific competition occurred between Moldavian balm and fenugreek as a result of increasing crop density.

At all cropping patterns the Moldavian balm dry herbage yields in 2020 were greater than those in 2021 that may be related to higher precipitation in 2020 that has led to increase in growth and yield of Moldavian balm. After sole Moldavian balm, the highest dry herbage yield was obtained in Mb:F(4:2) cropping pattern, in which the proportion of Moldavian balm was higher than other cropping patterns. Also in dragonhead (*Dracocephalum moldavica*)-soybean (*Glycine max*) intercropping^33^ observed that crop yields decreased in replacement intercropping patterns compared with those in sole crops. In 2020 the dry herbage yield in AMF + NFB fertilizer treatment was not significantly different with CF treatment and in 2021 increased compared with that in CF treatment. The increase in dry herbage yield of Moldavian balm as a result of bio-fertilizers application could be attributed to facilitative effect of arbuscular mycorrhizal fungi on fenugreek N-fixation and N transfer to Moldavian balm as reported by Li et al.^6^. In arbuscular mycorrhizal inoculation treatment, the faba bean N fixation and transferring to the intercropped wheat (*Triticum turgidum* L*.*) increased by 20%^22^.

The essential oil contents in Mb:F(1:1) and (2:2) intercropping patterns (0.252 and 0.261%) increased compared with those in Mb:F (100:50) and sole Moldavian balm (0.228 and 0.232%). Rezaei-Chiyaneh et al.^34^ reported that the essential oil contents in all intercropping patterns of fennel (*Foeniculum valgare*, Mill.)-common bean, were higher than that of sole fennel. The essential oil content in AMF + NFB fertilizer treatment was higher than those of CF and AMF fertilizer treatments. Since essential oil is one of the secondary metabolites, its production is influenced by nutrients availability and photosynthesis rate^35^. Increasing the content of essential oil with using AMF + NFB bio-fertilizer treatment could be attributed to improvement of plant growth characteristics and essential oil-forming glands due to the increase in the level of absorption and access to nutrients^36^. It seems that the intercropping of Moldavian balm with fenugreek especially in 1:1 and 2:2 patterns has increased the essential oil content by providing the main essential nutrients such as nitrogen which is affecting the activity of photosynthetic enzymes^37^ and essential oil content. On the other hand, using the bio-fertilizers improves the availability of macro- and micro-nutrients, as a result, affect the amount of essential oil^38^. Also, Kapoor et al.^39^ concluded that the use of bio-fertilizers caused changes in concentration of plant phytohormones which increased the formation of essential oil secreting glands and ultimately led to the production of more secondary metabolites. Increasing the content of essential oil due to use of bio-fertilizers in medicinal plants such as fennel^37^ and Moldavian balm^2,9^ has been specified.

The interaction effect of cropping pattern × fertilizer treatment (Fig. 9) on essential oil yield showed that the greatest values were observed in Mb:F(4:2) intercropping pattern, which can be attributed to the high proportion of Moldavian balm led to higher dry herbage yield in this cropping pattern. At all intercropping patterns except the Mb:F(1:1), the essential oil yield in AMF + NFB and CF treatments were not significantly different. Weisany et al.^40^ also found that in coriander-soybean intercropping, application of arbuscular mycorrhizal fungi (*Glomus intraradices*) significantly increased the essential oil yield of coriander. Vafadar-Yengeje et al.^2^ reported that in 1:1 faba bean- Moldavian balm intercropping pattern, the essential oil yield of Moldavian balm in chemical fertilizer and bio-fertilizer application was not significantly different. Also in previous studies increasing the essential oil yield of Moldavian balm^2,9,10^ and basil (*Ocimum basilicum* L.)^41^ as a result of bio-fertilizers application are specified. At all cropping patterns and fertilizer treatments the essential oil yield in 2020 were higher than those in 2021 that can be explained by higher precipitation in 2020 growth season which in turn increased the dry herbage yield, essential oil content and yield of Moldavian balm in this year (Table 5, Fig. 7 and 8).

Based on the results of GC–MS analysis, it was found that geranial, geranyl acetate, geraniol, neral and nerol were the main constituents of essential oil. In previous studies, it has been found that geraniol, geranial, geranyl acetate, neral and neryl acetate were the main constituents of essential oil in Moldavian balm^2,8,9,33^. Hussein et al.^42^ reported that in Moldavian balm the geranial and linalool were the main constituents of essential oil. The effects of fertilizer treatment and cropping pattern on major constituents of essential oil were different. The highest amount of geranial (61.4%) was observed in AMF treatment in sole Moldavian balm, which increased by 18.3 and 19.6% compared with that in AMF + NFB and CF treatments, respectively. Our results did not match with those of Vafadar-Yengeje et al.^2^ in intercropping of faba bean-Moldavian balm which reported that the highest geranial content was observed in chemical fertilizer treatment. The highest content of geraniol (26.2%) was related to the AMF treatment in the Mb:F(1:1) cropping pattern. Vafadar-Yengeje et al.^2^ found that in 1:1 faba bean-Moldavian balm intercropping the geraniol content was higher in bio-fertilizer and vermicompost treatments compared with chemical fertilizer. Also it has been found that the bio-fertilizers improved the content of geraniol in essential oil of Moldavian balm in comparison with control treatment^10^. The AMF + NFB fertilizer treatment in Mb:F(4:2) cropping pattern showed the highest content of geranyl acetate (47.1%). Karimzadeh Asl et al.^10^ also found that the greatest geranyl acetate content of Moldavian balm essential oil was obtained in bio-fertilizer treatment. On the other hand, the geranyl acetate content in CF fertilizer treatment of Mb:F(1:1) cropping pattern (31.2%) was higher than other cropping patterns. Also, the lowest contents of geranyl acetate in CF and AMF treatments were related to sole Moldavian balm (3.1 and 10.2%, respectively). In this regard, in faba bean-Moldavian balm intercropping, the content of geranyl acetate in chemical fertilizer treatment increased in all intercropping patterns compared to sole Moldavian balm^2^. Ganjewala and Luthra^43^ reported that a negative correlation was observed between the amount of geraniol and geranyl acetate in lemongrass (*Cymbopogon flexuosus* L.); so that as geraniol increased, the content of geranyl acetate decreased. They also reported that this correlation could be related to conversion of geranyl acetate to geraniol during the leaf growth stage.

It has been found that the differences in ecological factors, genetic origin and cropping patterns cause quantitative and qualitative differences in essential oil compounds^44^. In previous studies the positive effect of intercropping and use of bio-fertilizers has been reported on improving the essential oil composition of Moldavian Balm ^2,45^ and fennel^34^. Even though many studies indicated the positive effect of bio-fertilizer on essential oil constituents of medicinal plants such as white horehound (*Marrubium vulgare* L*.*)^46^ and Moldavian Balm^2,9,10^, on the other hand, a number of studies showed the advantageous effect of chemical fertilizers on the essential oil compositions^33^. Different biosynthesis pathways of these components may be responsible for different responses to fertilizer application^47^. The biosynthesis of essential oils is influenced by the amount of nutrients in the soil such as nitrogen and phosphorus^48^. It can be expected that the use of bio-fertilizers through the provision of more nutrients and creation and division of new essential oil-containing cells, glandular trichomes and essential oil channels increase essential oil in medicinal plants^16^. According to the results of present study, inoculation with AMF and NFB and intercropping with fenugreek could be used in order to improve the yield and quality of essential oil in Moldavian balm.

The LER is one of the intercropping evaluation indices that indicate the intercropping advantage in terms of land use. The results of LER values (Table 4) showed that the highest LER_Mb_ was obtained in the Mb:F(4:2) cropping pattern and CF fertilizer (0.87 and 0.85 in 2020 and 2021, respectively). Also, the highest LER_F_ values were obtained in Mb:F(100:50) cropping pattern and CF fertilizer treatment (0.93 and 0.98 in 2020 and 2021, respectively). In general, the partial LERs for fenugreek (LER_F_) were higher than those of Moldavian balm (LER_Mb_) (except for Mb:F(4:2) cropping pattern), that indicated the intercropping had more positive effect on fenugreek. Previous studies^49,50^ indicated that increasing the partial LER greater than 0.5 depends on the complementary degree of the intercropping components. Also, LER_T_ values higher than 1.0 indicate the superiority of intercropping over sole crop. Previous researches have shown that superiority in intercropping is due to different morphological properties and growth and the tendency of intercropping components to make optimum use of sources such as soil, moisture, light and nutrient elements^2,4,32,34^. Also there is a difference in the root structure, distribution of the canopy cover and the nutritional needs of the plants in intercropping^4,51^.

The highest LER_T_ (1.70) was obtained in Mb:F(100:50) cropping pattern and CF treatment in 2021, accounted for which was equivalent to an increase of 70% in land productivity compared to sole cropping of two species. The results of previous studies have also shown that when the legume species beside the other species are planted as intercropping, nitrogen fixation is stimulated due to the complementary effect, which increases the growth and yield of the legume species through increasing the number of active nodes^6,24,30^. Although the intercropping increases the competitiveness to absorb environmental resources, if one species has nitrogen fixation ability, competitive pressure will be reduced because the legume species will have less competition with other species in nitrogen absorption^4,51^. More distribution of nitrogen in the soil through nitrogen fixation and lower leaves fall of legumes improve soil fertility and adjacent plant growth in intercropping^24,30^. Fenugreek also appears to acidify the rhizosphere through nitrogen stabilization, thereby increasing the solubility and absorption of phosphorus by Moldavian balm^52^. At all cropping patterns the greatest essential oil yield of Moldavian balm was observed in AMF + NFB fertilizer treatment and the highest LER_T_ values were obtained in Mb:F(100:50) intercropping pattern. It seems that in our study the inoculation of AMF increased the N transfer from fenugreek to Moldavian balm as reported in maize (*Zea mays*) and alfalfa (*Medicago sativa*) intercropping system^53^. Also the nitrogen availability could be improved through inoculation of NFB which in turn increased the Moldavian balm growth and essential oil yield.

## Conclusions

The Moldavian balm height, LAI, dry herbage and essential oil yield were affected significantly by interaction effect of cropping pattern × fertilizer treatment. In Mb:F(1:1) and (100:50) intercropping patterns the highest dry herbage yields were observed in AMF + NFB fertilizer treatment. Among the fertilizer treatments the highest essential oil content (0.259%) was obtained in AMF + NFB treatment. At all cropping patterns except the Mb:F(1:1), the essential oil yields in AMF + NFB fertilizer treatments were not significantly different with those in CF treatments. Geranial, geranyl acetate, geraniol, neral and nerol were the main chemical constituents of Moldavian balm essential oil and affected differently by cropping patterns and fertilizer treatments. The LER_T_ indices in all intercropping patterns were greater than 1.0 in both years, which indicates more efficient use of land and resources in intercropping patterns than sole crop. In both years the LER_T_ values in Mb:F(100:50) intercropping patterns and AMF + NFB fertilizer treatments (1.55 and 1.63) were higher than those in other intercropping patterns and fertilizer treatments. Therefore, according to our findings, additive intercropping of Moldavian balm with fenugreek (Mb:F(100:50)) and the combined use of arbuscular mycorrhizal fungi + nitrogen-fixing bacteria (AMF + NFB) bio-fertilizers could be introduced as a eco-friendly and alternative methods to sole cropping and chemical fertilizer application in line with the goals of sustainable and cleaner production of Moldavian balm. For more studies, evaluating the other types of bio-fertilizers such as phosphorus-releasing bacteria and plant growth-promoting rhizobacteria (PGPR) could be recommended. Also there is a need for investigating the effects of intercropping of Moldavian balm with other legume and non-legume crops in different cropping systems in order to improve the efficiency and productivity of intercropping.

## Material and methods

### Experimental site

This research was conducted in Maragheh city in East Azarbaijan province, Iran (latitude 37˚4 N, longitude 46˚26 E, altitude 1478 m above sea level) in 2020 and 2021 growth seasons. The climatic data of monthly total precipitation and mean temperature of the experimental site during the growth seasons of 2020 and 2021 are presented in Table 5. The soil physicochemical characteristics of the experimental field at a depth of 0–30 cm are presented in Table 6.

**Table 6:** Physicochemical properties of the soil of experimental area in depth of 0–30 cm.

Parameter	Soil texture			pH	Organic matter (%)	EC (dS m^−1^)	Total N (%)	P (mg kg^−1^)	K (mg kg^−1^)
	Sandy loam								
	Clay (%)	Silt (%)	Sand (%)						
Value	9	27	64	7.28	1.1	1.56	0.033	8.5	586

### Experimental procedure

The factorial experiments were carried out based on randomized complete block design with three replications in 2020 and 2021. The cropping pattern (first factor) including Moldavian balm sole cropping (Mb), fenugreek sole cropping (F) and replacement intercropping ratios including 1 row of Moldavian balm + 1 row of fenugreek (Mb:F(1:1)), 2 rows of Moldavian balm + 2 rows of fenugreek (Mb:F(2:2)), and 4 rows of Moldavian balm + 2 rows of fenugreek (Mb:F(4:2)) and additive intercropping of Moldavian balm + fenugreek Mb:F(100: 50) (100% optimum density of Moldavian balm + 50% optimum density of fenugreek planted between Moldavian balm rows). The fertilizer treatment (second factor) was consisted of 100% chemical fertilizer (CF), application of arbuscular mycorrhizal fungi (AMF) and combined application of AMF and nitrogen-fixing bacteria (AMF + NFB).

The CF treatment was 50 kg ha^−1^ urea and 80 kg ha^−1^ triple superphosphate (according to soil test results); which were used at planting time. Myco-Root bio-fertilizer contains arbuscular mycorrhiza fungi (AMF) of *Glomus mosseae*, *Glomus intraradices* and *Glomus etunicatum* with count 10^7^ to 10^8^ CFU/gr is provided by Zist Fanavar Pishtaz Varian Company, Karaj, Iran. This bio-fertilizer is an easy-to-use powder form that is used for crops as seed inoculation. According to the manufacturer's instructions, the seeds of Moldavian balm and fenugreek required for the experimental area were placed in the shade on nylon or a clean surface, and after spraying a small amount of water on them, the required amount of AMF bio-fertilizer was added and mixed thoroughly; so that all the seeds were covered with a uniform layer of bio-fertilizer and after drying the seeds in shade, the planting was done. In order to inoculation of NFB, Biofarm bio-fertilizer contains *Azospirillum* and *Azotobacter* bacteria with a population of 2 × 10^7^ CFU/gr is provided by Nature Biotechnology Company (Biorun) Karaj, Iran. Similar to the Myco-Root, the seeds of the Moldavian balm and fenugreek were inoculated with Biofarm and then planted.

The Moldavian balm and fenugreek were planted with densities of 32 and 50 plants m^−2^, respectively. Both crops were planted with 25 cm row space on 04 May 2020 and 15 May 2021. The size of the experimental plots in sloe Moldavian balm, sole fenugreek, 1 row of Moldavian balm + 1 row of fenugreek (Mb:F(1:1)), 2 rows of Moldavian balm + 2 rows of fenugreek (Mb:F(2:2)) and additive intercropping of Moldavian balm + fenugreek Mb:F(100: 50) were 3 m (12 rows) wide × 3 m long. In intercropping of 4 rows of Moldavian balm + 2 rows of fenugreek (Mb:F(4:2)) the size of the experimental plot was 4 m (16 rows) wide × 3 m long. The furrow irrigation was done after planting of both crops with 5-day intervals. The weeds in plots were removed by hand weeding.

### Ethical approval

This experimental research upon plants complies with relevant institutional, national, and international guidelines and legislation. Moldavian balm and fenugreek seeds used in experiment, were purchased from Tabriz, Iran.

### Plant height

In both years in order to measure the height of Moldavian balm, at flowering stage (on 29 July 2020 and 09 August 2021) 10 plants from each plot were randomly selected after removing the marginal effects (side rows and half a meter from the sides of the middle rows) and plant height was measured.

### Leaf area index (LAI)

In both years the LAI was determined at flowering stage (on 29 July 2020 and 09 August 2021). In order to measure the leaf area of Moldavian balm, the plants in 1 m^−2^ area of each plot were cut from the soil surface, transferred to the laboratory and the leaf area of the all plants were measured by Delta-T leaf area meter (Delta-T Devices, Cambridge, England). By dividing the leaf area to the ground area (1 m^−2^) the LAI was obtained for all treatments^54^.

### Moldavian balm dry herbage yield and fenugreek grain yield

Moldavian balm plants were harvested manually from each plot (1 m^−2^ area) at the flowering stage to determine dry herbage yield on 29 July 2020 and 09 August 2021, and after drying in shade and room temperature (for 7 days), the herbage yield was measured as kg ha^−1^ ^2^. Fenugreek plants were harvested manually from each plot (1 m^−2^ area) at maturity stage on 02 September 2020 and 08 September 2021. In order to obtain the grain yield per unit area the harvested plants were dried at 30 °C for 48 h and after hand-threshing the grain yield was measured.

### The content and yield of essential oil

In flowering stage the Moldavian balm herbage was harvested, dried and powdered. Then 100 g from powder was hydrodistilled for 3.0 h by a Clevenger device^9,55^. In order to dehydrate the obtained essential oil, the anhydrous sodium sulphate was used; and by measuring the amount of essential oil, it was expressed as a percentage (EO content). The samples were stored at 4 °C until GC–MS analysis. Also, the yield of essential oil (kg ha^−1^) was calculated as follows^8^.

$${\text{Essential oil yield }}\left( {{\text{kg ha}}^{{ - {1}}} } \right) = {\text{Essential oil content }}\left( \% \right) \times {\text{Herbage yield }}\left( {{\text{kg ha}}^{{ - {1}}} } \right)$$

### Gas chromatography-mass spectrometry

Gas chromatography-mass spectrometry (GC–MS) analysis was done on an Agilent 6890N gas chromatograph with a 5973 mass-selective detector (Agilent Technologies, CA, USA) as described by Vafadar-Yengeje et al.^2^. Identification of compounds was carried out by comparison of the mass spectra of each component either with those stored in the Wiley 7.0 and Adams mass spectral-retention index libraries^56^.

### LER assessment

In Moldavian balm-fenugreek intercropping patterns the LER values were assessed as follows^2^ as:

1 $${\text{LER}}_{{{\text{Mb}}}} = {\text{ Y}}_{{{\text{MbI}}}} /{\text{Y}}_{{{\text{Mb}}}} {\text{andLER}}_{{\text{F}}} = {\text{ Y}}_{{{\text{FI}}}} /{\text{ Y}}_{{\text{F}}}$$

2 $${\text{LER}}_{{\text{T}}} = {\text{ LER}}_{{{\text{Mb}}}} + {\text{ LER}}_{{\text{F}}}$$

Where Y_Mb_ and Y_MbI_ are the Moldavian balm dry herbage yields in sole cropping and intercropping, respectively, and Y_F_ and Y_FI_ are the fenugreek grain yields in sole cropping and intercropping patterns, respectively. Also, LER_Mb_ and LER_F_ represent the partial LER of Moldavian balm and fenugreek, respectively, and LER_T_ is the total LER. When LER_T_ is equal to 1.0, indicates the same competitive ability of both crops in intercropping. This situation occurs if the inter-specific competition is equal to the intra-species competition in the intercropping components. In addition, if the increase in the yield of one crop is accompanied by a decrease in the yield of another crop in intercropping, the LER_T_ value will be equal to 1.0. If the value of LER_T_ is less than 1.0, sole crop is superior to intercropping, and if the value of LER_T_ is higher than 1.0, intercropping is more beneficial than sole crop ^2,4^.

### Statistical analysis

For analysis of variance (ANOVA) the SAS Version 9.0.3 was used. For two growing seasons of 2020 and 2021 and all traits the combined ANOVA was done based on complete randomized block design with 15 treatments and three replicates. The data of LER_M_, LER_F_, and LER_T_ were not subjected to analysis of variance. The experimental data met the assumptions of normality and variance homogeneity and no transformation was needed. For comparison of the means the Duncan´s multiple range test was used at *p* ≤ 0.05.

## Data Availability

The necessary information is available from the corresponding author on reasonable request.

## References

[1] Kassam A, Brammer H (2013). Combining sustainable agricultural production with economic and environmental benefits. Geogr. J..

[2] Vafadar-Yengeje L, Amini R, Nasab ADM (2019). Chemical compositions and yield of essential oil of Moldavian balm (*Dracocephalum moldavica* L.) in intercropping with faba bean (*Vicia faba* L.) under different fertilizers application. J. Clean. Prod..

[3] Vrignon-Brenas S, Celette F, Piquet-Pissaloux A, Jeuffroy MH, David C (2016). Early assessment of ecological services provided by forage legumes in relay intercropping. Eur. J. Agron..

[4] Amini R, Choubforoush Khoei B, Dabbagh Mohammadi Nasab A, Raei Y (2020). Effects of intercropping sugar beet (*Beta vulgaris* L.) with millet, soybean and Moldavian balm on yield and quality in an organic production system. Biol. Agric. Hortic..

[5] Calabi-Floody M, Medina J, Rumpel C, Condron LM, Hernandez M, Dumont M, de La Luz Mora M (2018). Smart fertilizers as a strategy for sustainable agriculture. Adv. Agron..

[6] Li M, Hu J, Lin X (2022). The roles and performance of arbuscular mycorrhizal fungi in intercropping systems. Soil Ecol. Lett..

[7] Abd El-Baky HH, El-Baroty GS (2007). Chemical and biological evaluation of the essential oil of Egyptian Moldavian balm. Global J. Biotech. Biochem..

[8] Amini R, Ebrahimi A, Dabbagh Mohammadi Nasab A (2020). Moldavian balm (*Dracocephalum moldavica* L.) essential oil content and composition as affected by sustainable weed management treatments. Ind. Crops Prod..

[9] Amini R, Zafarani-Moattar P, Shakiba MR, Sarikhani MR (2020). Essential oil yield and composition of Moldavian balm (*Dracocephalum moldavica* L.) as affected by inoculation treatments under drought stress condition. J. Essent. Oil Bear. Pl..

[10] Karimzadeh Asl K, Ghorbanpour M, Marefatzadeh Khameneh M, Hatami M (2018). Influence of drought stress, biofertilizers and zeolite on morphological traits and essential oil constituents in *Dracocephalum moldavica* L. J. Med. Plants..

[11] Irankhah S, Ganjeali A, Mashreghi M, Lari Z (2021). Mixed inoculum of rhizobacteria and arbuscular mycorrhizal fungus enhance diosgenin contain and phosphorus uptake in fenugreek under drought stress. Rhizosphere..

[12] Riasat M, Pakniyat H, Heidari B, Jafari AA (2018). Variations in phytophenol compounds in association with morphological traits in Trigonella spp. Accessions. Annu. Res. Rev. Biol..

[13] Osman HE, Al-Jabri M, El-Ghareeb DK, Al-Maroai YA (2020). Impact of aluminum and zinc oxides on morphological characters, germination, metals accumulation and DNA in fenugreek (*Trigonella foenum-graecum*). J. Saudi Soc. Agric. Sci..

[14] Wani SA, Kumar P (2016). Fenugreek enriched extruded product: optimization of ingredients using response surface methodology. Int. Food Res. J..

[15] Duchene O, Vian JF, Celette F (2017). Intercropping with legume for agroecological cropping systems: Complementarity and facilitation processes and the importance of soil microorganisms. Agric. Ecosyst. Environ..

[16] Rostaei M, Fallah S, Lorigooini Z, Surki AA (2018). Crop productivity and chemical compositions of black cumin essential oil in sole crop and intercropped with soybean under contrasting fertilization. Ind. Crops Prod..

[17] Yang F, Huang S, Gao R, Liu W, Yong T, Wang X, Wu X, Yang W (2014). Growth of soybean seedlings in relay strip intercropping systems in relation to light quantity and red: far-red ratio. Field Crops Res..

[18] Machiani MA, Javanmard A, Morshedloo MR, Maggi F (2018). Evaluation of competition, essential oil quality and quantity of peppermint intercropped with soybean. Ind. Crops Prod..

[19] Brundrett MC, Tedersoo L (2018). Evolutionary history of mycorrhizal symbioses and global host plant diversity. New Phytol..

[20] Dieng A, Duponnois R, Ndoye I, Baudoin E (2017). Positive feedback with mycorrhizal fungi alleviates negative effects of intercropping the energy crop *Jatropha curcas* with *Crotalaria retusa*. Symbiosis.

[21] Barea, J.M., Werner, D., Azcon-Guilar, & C., Azcon, R. Interactions of arbuscular mycorrhiza and nitrogen- fixing symbiosis in sustainable agriculture. In: Werner, D., Newton, W.E. (eds.), Nitrogen Fixation in Agriculture, Forestry, Ecology, and the Environment. Dordrecht: Springer, 199–222. (2005).

[22] Ingraffia R, Amato G, Frenda AS, Giambalvo D (2019). Impacts of arbuscular mycorrhizal fungi on nutrient uptake, N_2_ fixation, N transfer, and growth in a wheat/faba bean intercropping system. PLoS ONE.

[23] Bushra ZI, Hussain A, Dar A, Ahmad M, Wang X, Brtnicky M, Mustafa A (2022). Combined use of novel endophytic and rhizobacterial strains upregulates antioxidant enzyme systems and mineral accumulation in wheat. Agronomy.

[24] Płaza A, Niewiadomska A, Górski R, Rudzinski R, Rzazewska E (2022). The effect of the nitrogen-fixing bacteria and companion red clover on the total protein content and yield of the grain of spring barley grown in a system of organic agriculture. Agronomy.

[25] Den Hollander NG, Bastiaans L, Kropff MJ (2007). Clover as a cover crop for weed suppression in an intercropping design: II. Competitive ability of several clover species. Eur. J. Agron..

[26] Ebwongu M, Adipala E, Ssekabembe CK, Kyamanywa S, Bhagsari AS (2001). Effect of intercropping maize and solanum potato on yield of the component crops in central Uganda. Afr. Crop Sci. J..

[27] Geren H, Avcioglu R, Soya H, Kir B (2008). Intercropping of corn with cowpea and bean: Biomass yield and silage quality. Afr. J. Biotechnol..

[28] Agegnehu G, Ghizaw A, Sinebo W (2006). Yield performance and land-use efficiency of barley and faba bean mixed cropping in Ethiopian highlands. Eur. J. Agron..

[29] Baum C, El-Tohamy W, Gruda N (2015). Increasing the productivity and product quality of vegetable crops using arbuscular mycorrhizal fungi: A review. Sci. Hortic..

[30] Zhao M, Jones CM, Meijer J, Lundquist PO, Fransson P, Carlsson G, Hallin S (2017). Intercropping affects genetic potential for inorganic nitrogen cycling by root-associated microorganisms in *Medicago sativa* and *Dactylis glomerata*. Appl. Soil Ecol..

[31] Yavas I, Unay A (2016). Evaluation of physiological growth parameters of maize in maize legume intercropping system. J. Anim. Plant Sci..

[32] Reddy, M. S., & Willey, R. W. (2008). A study of pearl millet\\groundnut intercropping with particular emphasus on the efficiencies of leaf canopy and rooting pattern. *Embrapa Semiárido-Folderes/Folhetos/Cartilhas (INFOTECA-E)* (2008).

[33] Fallah S, Rostaei M, Lorigooini Z, Surki AA (2018). Chemical compositions of essential oil and antioxidant activity of dragonhead (*Dracocephalum moldavica*) in sole crop and dragonhead-soybean (*Glycine max*) intercropping system under organic manure and chemical fertilizers. Ind. Crops Prod..

[34] Rezaei-Chiyaneh E, Amirnia R, Machiani MA, Javanmard A, Maggi F, Morshedloo MR (2020). Intercropping fennel (*Foeniculum vulgare* L.) with common bean (*Phaseolus vulgaris* L.) as affected by PGPR inoculation: A strategy for improving yield, essential oil and fatty acid composition. Sci. Hortic..

[35] Rowan DD (2011). Volatile metabolites. Metabolites.

[36] Copetta A, Lingua G, Berta G (2006). Effects of three AM fungi on growth, distribution of glandular hairs, and essential oil production in *Ocimum basilicum* L. var. Genovese. Mycorrhiza.

[37] El-Azim A, Khater WM, Badawy RMR (2017). Effect of bio-fertilization and different licorice extracts on growth and productivity of *Foeniculum vulgare*. Mill. Middle East J. Agric. Res..

[38] Singh JS, Pandey VC, Singh DP (2011). Efficient soil microorganisms: A new dimension for sustainable agriculture and environmental development. Agric. Ecosyst. Environ..

[39] Kapoor R, Anand G, Gupta P, Mandal S (2017). Insight into the mechanisms of enhanced production of valuable terpenoids by arbuscular mycorrhiza. Phytochem. Rev..

[40] Weisany W, Tahir NAR, Schenk PM (2021). Coriander/soybean intercropping and mycorrhizae application lead to overyielding and changes in essential oil profiles. Eur. J. Agron..

[41] Tahami MK, Jahan M, Khalilzadeh H, Mehdizadeh M (2017). Plant growth promoting rhizobacteria in an ecological cropping system: A study on basil (*Ocimum basilicum* L.) essential oil production. Ind. Crops Prod..

[42] Hussein MS, El-Sherbeny SE, Khalil MY, Naguib NY, Aly SM (2006). Growth characters and chemical constituents of *Dracocephalum moldavica* L. plants in relation to compost fertilizer and planting distance. Sci. Hortic..

[43] Ganjewala D, Luthra R (2009). Geranyl acetate esterase controls and regulates the level of geraniol in lemongrass (*Cymbopogon flexuosus* Nees ex Steud.) mutant cv. GRL-1 leaves. Z. Naturforsch C..

[44] Argyropoulou C, Daferera D, Tarantilis PA, Fasseas C, Polissiou M (2007). Chemical composition and variation during development of the essential oil from leaves of *Lippia citriodora* HBK (Verbenaceae). Biochem. Syst. Ecol..

[45] Faridvand S, Rezaei-Chiyaneh E, Battaglia ML, Gitari HI, Raza MA, Siddique KH (2022). Application of bio and chemical fertilizers improves yield, and essential oil quantity and quality of Moldavian balm (*Dracocephalum moldavica* L.) intercropped with mung bean (*Vigna radiata* L.). Food Energy Secur..

[46] El-Leithy AS, El-Hanafy SH, Omer EA, El-Sayed AAA (2013). Effect of nitrogen and potassium biofertilization on growth yield and essential oil production of white horehound *Marrubium vulgare* L. J. Hortic. Sci. Ornam. Plants..

[47] Sangwan NS, Farooqi AHA, Shabih F, Sangwan RS (2001). Regulation of essential oil production in plants. Plant Growth Regul..

[48] Koeduka T, Fridman E, Gang DR, Vassão DG, Jackson BL, Kish CM, Orlova I, Spassova SM, Lewis NG, Noel JP, Baiga TJ (2006). Eugenol and isoeugenol, characteristic aromatic constituents of spices, are biosynthesized via reduction of a coniferyl alcohol ester. Proc. Natl. Acad. Sci..

[49] Monti M, Pellicanò A, Santonoceto C, Preiti G, Pristeri A (2016). Yield components and nitrogen use in cereal-pea intercrops in Mediterranean environment. Field Crop Res..

[50] Hauggaard-Nielsen H, Jørnsgaard B, Kinane J, Jensen ES (2008). Grain legume cereal intercropping: The practical application of diversity, competition and facilitation in arable and organic cropping systems. Renew. Agric. Food Syst..

[51] Karpenstein-Machan M, Stuelpnagel R (2000). Biomass yield and nitrogen fixation of legumes monocropped and intercropped with rye and rotation effects on a subsequent maize crop. Plants Soil..

[52] Goh CH, Nicotra AB, Mathesius U (2016). The presence of nodules on legume root systems can alter phenotypic plasticity in response to internal nitrogen independent of nitrogen fixation. Plant Cell Environ..

[53] Zhang H, Wang X, Gao Y, Sun B (2020). Short-term N transfer from alfalfa to maize is dependent more on arbuscular mycorrhizal fungi than root exudates in N deficient soil. Plant Soil..

[54] Amini R, Alizadeh H, Yousefi A (2014). Interference between red kidneybean (*Phaseolus vulgaris* L.) cultivars and redroot pigweed (*Amaranthus retroflexus* L.). Eur. J. Agron..

[55] Pharmacopoeia, B. HMSO, vol. 2, *A137eA138* (London 1988).

[56] Adams RP (2017). Identification of Essential Oil Components by Gas Chromatography/Mass Spectrometry.

